# Pyranoxanthones from *Mesua ferrea*

**DOI:** 10.3390/molecules16075647

**Published:** 2011-06-30

**Authors:** Soek Sin Teh, Gwendoline Cheng Lian Ee, Mawardi Rahmani, Yun Hin Taufiq-Yap, Rusea Go, Siau Hui Mah

**Affiliations:** 1 Department of Chemistry, Faculty of Science, Universiti Putra Malaysia, 43400 Serdang, Selangor, Malaysia; 2 Department of Biology, Faculty of Science, Universiti Putra Malaysia, 43400 Serdang, Selangor, Malaysia

**Keywords:** mesuaferrin A, mesuaferrin B, pyranoxanthone, *Mesua ferrea*, Guttiferae

## Abstract

Our phyto chemical studies on the root bark extracts of *Mesua ferrea* have led to the isolation of two new pyranoxanthones, mesuaferrin A (**1**) and mesuaferrin B (**2**). Five other compounds – caloxanthone C (**3**), 1,8-dihydro-3-methoxy-6-methylanthraquinone (**4**), *β*-sitosterol (**5**), friedelin (**6**) and betulinic acid (**7**) – were also successfully isolated. Structural elucidations of these compounds were achieved using 1D and 2D NMR and MS techniques.

## 1. Introduction

**Figure 1:**
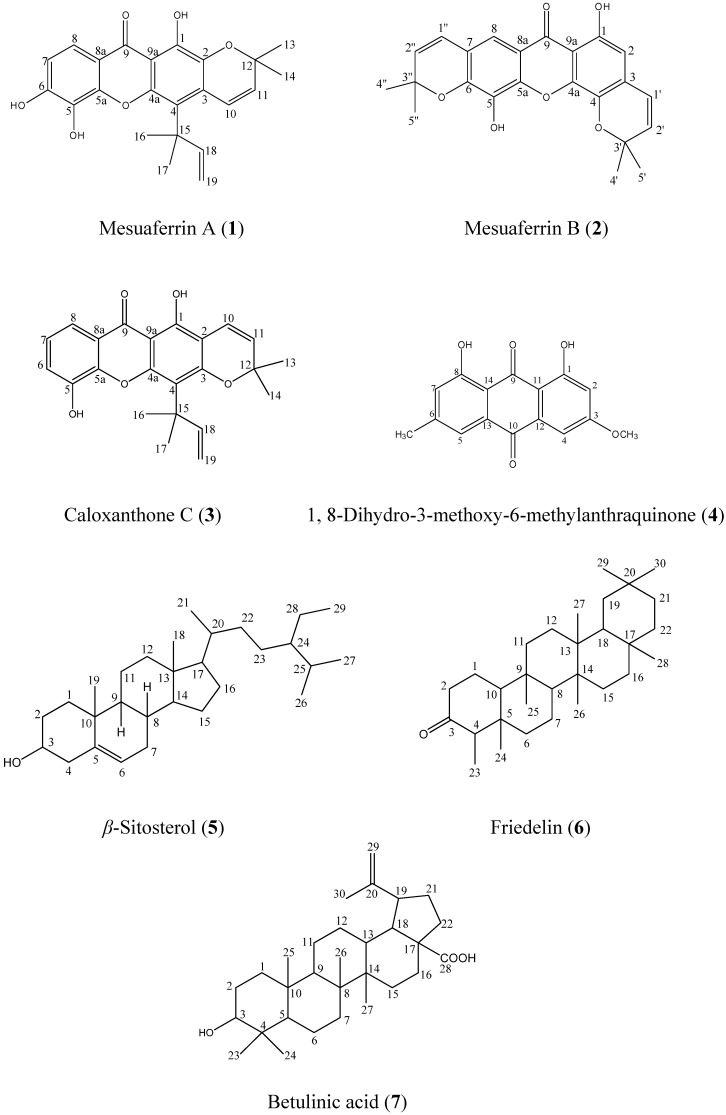
Structures of compounds.

*Mesua ferrea* (Clusiaceae) is a large evergreen tree, usually found growing in evergreen tropical to semi-tropical forests. It is used in folk medicine for the treatment of fevers, dyspepsis and renal diseases [1]. Some phytochemical investigations on secondary metabolites from *Mesua sp.* have revealed the existence of xanthones, coumarins, biflavonoids and triterpenoids [2,3]. Our ongoing research on *Mesua ferrea* has now identified two new pyranoxanthones – mesuaferrin A (**1**) and mesuaferrin B (**2**) – in the root bark of *Mesua ferrea* (Figure 1). We report here the isolation and structural elucidation of these two new compounds. 

## 2. Results and Discussion

The new compound, mesuaferrin A (**1**) which is a yellowish crystalline material with melting point of 180–181 °C was isolated from the hexane extract of *Mesua ferrea*. The EIMS spectrum gave a molecular ion (M^+^) peak at *m/z* 394, while the HRESIMS spectrum gave 394.1320 (calc’d. 394.1417), consistent with the molecular formula C_23_H_22_O_6_. The IR spectrum showed ν_max_ at 3334 (OH), 2965 (CH_3_), 2926 (CH_2_), 1582 (C=O), 1260 and 1185 (aromatic C=C) cm^−1^. Maximum absorptions of UV spectrum were observed at 337, 290, 283, 243 and 207 nm, suggesting a xanthone skeleton. The ^13^C and DEPT NMR spectra of compound **1** revealed the presence of twelve quaternary carbons (δ 41.5, 78.3, 103.4, 105.7, 113.2, 113.8, 131.1, 144.6, 149.1, 154.2, 156.8 and 159.0), five methines (δ 112.9, 116.2, 117.6, 127.3 and 156.9), one methylene (δ 103.4) and four methyls (δ 28.0 × 2 and 28.3 × 2). The carbonyl group of the xanthone skeleton was confirmed by the existence of a low field signal at δ 180.3. The ^1^H and COSY NMR spectra showed the presence of a chelated hydroxyl group (δ 13.53, 1H, s), two pairs of ortho-coupled protons (δ 6.94 & 7.68, 1H each, d, *J* = 8.8 Hz; δ 5.61 & 6.76, 1H each, d, *J* = 10.1 Hz) and four methyl proton signals with overlapped chemical shifts (δ 1.51 × 2, 1.64 × 2, 6H each signal, s). The existence of a pyrano ring and its attachment to the main xanthone skeleton at C-2 and C-3 was accounted for by the two olefinic coupled protons H-10 and H-11 showing HMBC linkages to δ 105.7 (C-3) and 159.0 (C-2).

**Figure 2:**
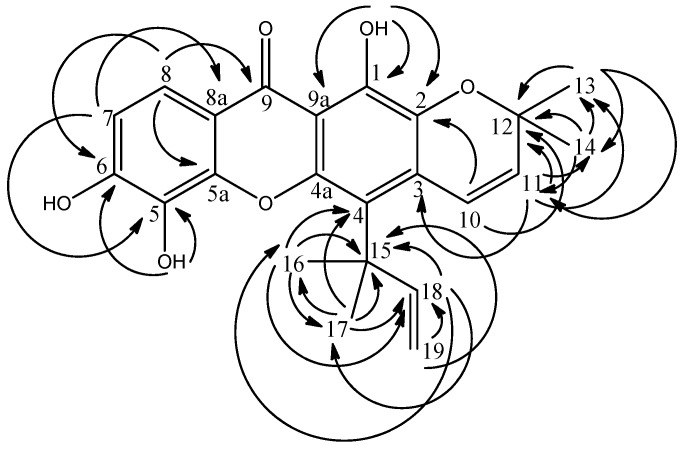
HMBC^ 2^*J* and ^3^*J* correlations between ^1^H and ^13^C in **1**.

Meanwhile, characteristic prenyl signals at δ 1.64 (16-CH_3_, 17-CH_3_), 5.04 (H-19b), 5.21 (H-19a), and 6.73 (H-18) were observed. *Trans* coupling between the doublet at δ 5.21 and the doublet of doublet at δ 6.73 and *cis* coupling between the doublet at δ 5.04 and the doublet of doublet at δ 6.73 accounts for the double bond in the prenyl moiety. A ^3^*J* HMBC correlation between the two methyls and δ 113.2 assigns the prenyl to C-4 (δ 113.2). The pair of *ortho* coupled protons (H-8 and H-7) and the *cis* olefinic protons (H-10 and H-11) had their chemical shifts rightfully assigned through long-range correlations with δ 113.8 & 131.1 (C-8a and C-5) and 105.7 and 159.0 (C-3 and C-2) respectively. HMBC correlations are shown in Figure 2. Hence, the structure of compound **1** was elucidated to be 1,5,6-trihydroxy-4-(3’,3’-dimethyl-propenyl)-6”,6”-dimethylpyrano-[2”,3”:2,3]-xanthone and named mesuaferrin A. The NMR data of this compound are summarized in Table 1.

**Table 1:** ^1^H-NMR (500 MHz, CDCl_3_) and ^13^C-NMR (125 MHz, CDCl_3_) data for mesuaferrinA (**1**) and mesuaferrin B (**2**). 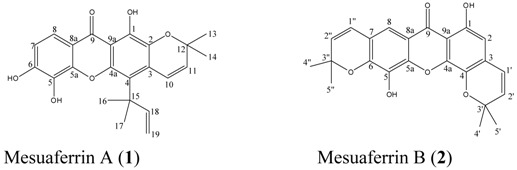

Mesuaferrin A (1)			Mesuaferrin B (2)		
Position	^1^H( *δ*)	^13^C( *δ*)	Position	^1^H( *δ*)	^13^C( *δ*)
1	-	156.8	1	-	157.8
2	-	159.0	2	6.42 ( *s*)	95.4
3	-	105.7	3	-	104.9
4	-	113.2	4	-	160.6
4a	-	154.2	4a	-	156.9
5	6.27 ( *s*)	131.1	5	-	132.2
5a	-	144.6	5a	-	145.2
6	-	149.1	6	-	144.8
7	6.94 ( *d*, 8.3)	112.9	7	-	117.9
8	7.68 ( *d*, 9.2)	117.6	8	7.47 ( *s*)	113.6
8a	-	113.8	8a	-	114.8
9	-	180.9	9	-	180.3
9a	-	103.4	9a	-	103.3
10	6.76 ( *d*, 10.1)	116.2	1’	6.72 ( *d*, 10.1)	115.5
11	5.61 ( *d*, 10.1)	127.3	2’	5.59 ( *d*, 10.1)	127.6
12	-	78.3	3’	-	78.3
13	1.51( *s*)	28.0	4’	1.47 ( *s*)	28.5
14	1.51( *s*)	28.0	5’	1.47 ( *s*)	28.5
15	-	41.5	1”	6.43 ( *d*, 10.1)	121.5
16	1.64 ( *s*)	28.3	2”	5.73 ( *d*, 10.1)	131.1
17	1.64 ( *s*)	28.3	3”	-	79.0
18	6.73 ( *dd*)	156.9	4”	1.53 ( *s*)	28.6
19a	5.21 ( *d*, 19.2)	103.4	5”	1.53 ( *s*)	28.6
19b	5.04 ( *d*, 11.9)	103.4	-	-	-
1-OH	13.53 ( *s*)	-	1-OH	13.11 ( *s*)	-
5-OH	6.26 ( *s*)	-	5-OH	5.58 ( *s*)	-
6-OH	-	-	-	-	-

**Figure 3:**
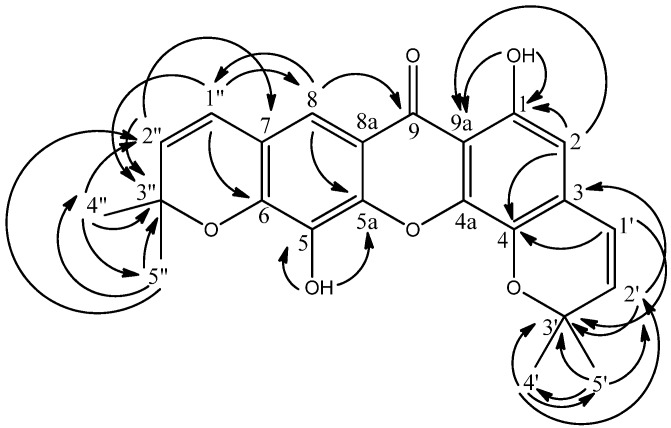
HMBC^ 2^*J* and ^3^*J* correlations between ^1^H and ^13^C in **2**.

The second new compound, mesuaferrin B (**2**), was isolated from the dichloromethane extract of *Mesua ferrea* as yellowish crystals with a melting point of 245–246 °C. The EIMS spectrum gave a molecular ion (M^+^) peak at *m/z* 392 while the HRESIMS spectrum gave 392.1148 (calc’d 392.1260), consistent with the molecular formula C_23_H_20_O_6_. The IR spectrum showed ν_max_ at 3426 (OH), 2925 (CH_3_), 2857 (CH_2_), 1739 (C=O), 1640 and 1602 (C=C aromatic) cm^−1^. Maximum absorptions of UV spectrum were observed at 345, 298, 290 and 208 nm suggesting a xanthone skeleton. The ^1^H-NMR and COSY spectra of compound **2** indicated the presence of two pyrano rings flanking the main xanthone skeleton. This is in contrast to compound **1** which carries only one pyrano ring. The two pyrano rings were rightfully assigned and shown to be attached to C-3, C-4 and C-6, C-7 by HMBC correlations between these carbons and the two pairs of *cis* olefinic hydrogens. H-2’ (δ 5.59) gave a ^3^*J* correlation with C-3 (δ 104.9) while H-1’ (δ 6.72) correlated to C-4 (δ 160.6) via a ^3^*J* correlation. This positions the first pyrano ring at C-3 and C-4. The second pyrano ring, on the other hand was deduced to be attached to C-6 and C-7 by evidence of H-1” and H-2” showing ^3^*J* correlations to C-6 (δ 144.8) and C-7 (δ 117.9) respectively (see Figure 3). The two aliphatic methyls at δ 1.47 (C-4’ and C-5’) gave ^2^*J* and ^3^*J* connectivities to δ 78.3 (C-3’) and δ 127.6 (C-2’). Also, long-range correlations between the pair of *cis* olefinic protons at δ 6.72 and 5.59 (H-1’ and H-2’) and the quaternary carbon at δ 78.3 (C-3’) was observed. Similarly, the HMBC spectra demonstrated the correlations between δ 1.53 (H-4” and H-5”) to δ 79.0 (C-3”) while the two *cis* olefinic protons H-1” and H-2” (δ 6.43 and 5.73) gave cross peaks to δ 79.0 (C-3”). In addition, two lone aromatic protons (δ 6.42, 1H, s and δ 7.47, 1H, s), a free hydroxy group (δ 5.58, 1H, s) and a chelated hydroxy group (δ 13.11, 1H, s) were also observed in compound **2**. These protons were assigned using their HMBC correlations as shown in Figure 3. 

The ^13^C and DEPT NMR spectra showed the presence of twelve quaternary carbons (δ 78.3, 79.0, 103.3, 104.9, 114.8, 117.9, 132.2, 144.8, 145.2, 156.9, 157.8 and 160.6), six methines (δ 95.4, 113.6, 115.5, 121.5, 127.6 and 131.1) and two methyls [δ 28.5 (CH_3_ × 2) and 28.6 (CH_3_ × 2)]. 

The free hydroxyl group signal at δ 5.58 has connectivity with δ 132.2 and 145.2 in the HMBC spectrum. The latter signal was assigned at C-5a as it was correlated to the aromatic proton at δ 7.47. Therefore, δ 5.52 and δ 132.2 were assigned as H-5 and C-5. Further analysis of HMQC and HMBC spectra implied the aromatic proton at δ 6.42 has ^1^*J* connectivity with δ 95.4 and long-range correlation with δ 160.6 (C-4). As a result, δ 95.4 was assigned to C-2.

Hence, the structure of compound **2** was elucidated to be 1,5-dihydroxy-6’,6’-dimethylpyrano-[2’,3’:4,3]-6”,6”-dimethylpyrano-[2”,3”:6,7]-xanthone and named mesuaferrin B. The NMR data of this compound are summarized in Table 1. 

The known compounds caloxanthone C (**3**), friedelin (**6**) and β-sitosterol (**5**) were isolated from the hexane extract and 1,8-dihydro-3-methoxy-6-methylanthraquinone (**4**) and betulinic acid (**7**) were isolated from the dichloromethane extract and identified by comparison of their spectral data with literature values, as indicated in the Experimental.

## 3. Experimental

### 3.1. General

Infrared spectra were measured using the universal attenuated total reflection (UATR) technique on a Perkin-Elmer 100 Series FT-IR spectrometer. EIMS were recorded on a Shimadzu GCMS-QP5050A spectrometer. NMR spectra were obtained using a Unity JEOL 500 MHz FTNMR spectrometer using CDCl_3_ as solvent and tetramethylsilane (TMS) as internal standard. Ultraviolet spectra were recorded in EtOH on a Shimadzu UV-160A, UV-Visible Recording Spectrophotometer.

### 3.2. Plant Material

The root bark of *Mesua ferrea* was collected from the Sri Aman district in Sarawak, Malaysia. The plant material was identified by Associate Professor Dr Rusea Go, Biology Department, Faculty of Science, Universiti Putra Malaysia.

### 3.3. Extraction and Isolation

The three kg of air-dried powdered sample was extracted successively with n-hexane and dichloromethane (5 L, 48 h). The extracts were dried under reduced pressure using a rotary evaporator to yield hexane (49.6 g) and dichloromethane (19.5 g) extracts. Each of these extracts was chromatographed over a silica gel column using a stepwise gradient system (hexane/chloroform, chloroform/ethyl acetate, and ethyl acetate/methanol). The column chromatography of the hexane extract gave mesuaferrin A (**1**, 20 mg), caloxanthone C (**3**, 25 mg), friedelin (**6**, 15 mg) and β-sitosterol (**5**, 24 mg), while the dichloromethane extract gave mesuaferrin B (**2**, 22 mg), 1,8- dihydro-3-methoxy-6-methyl-anthraquinone (**4**, 5 mg) and betulinic acid (**7**, 12 mg) 

### 3.4. Spectral Data

*Mesuaferrin A* (**1**): Yellow crystals, m.p. 180–181 °C. UV λ_max_ (EtOH) nm (log ε): 207 (5.21), 243 (5.28), 283 (5.35), 290 (5.36), 337 (5.42). IR ν_max_ (cm^−1^): 3334, 2965, 2926, 1582, 1260 and 1185. MS m/z (rel. int.): 394 [M^+^] (33), 380 (25), 379 (100), 351 (11), 182 (12), 162 (13), 153 (14), 55 (9). For ^1^H- and ^13^C- NMR spectra, see Table 1.

Mesuaferrin *B* (**2**): Yellow crystal, m.p. 245–246 °C. UV λ_max_ (EtOH) nm (log ε): 208 (5.21), 290 (5.36), 298 (5.37), 345 (5.43). IR ν_max_ (cm^−1^): 3426, 2925, 2857, 1739, 1640 and 1602. MS m/z (rel. int.): 392 [M^+^] (39), 378 (24), 377 (100), 361 (10), 347 (7), 203 (6), 181 (65). For ^1^H- and ^13^C- NMR spectra, see Table 1.

*Caloxanthone C* (**3**): Yellow crystals, m.p. 213–215 °C (Lit. 217 °C) [4]. Spectral data were consistent with the literature [4].

*1,8-Dihydro-3-methoxy-6-methylanthraquinone* (**4**): Orange needle-like crystals, m.p. 210–212 °C (lit. [5] 209–210 °C). Spectral data were consistent with the literature [5].

*β-Sitosterol* (**5**): White needles, m.p. 135 °C (lit. [6] 136–138 °C). Spectral data agreed with the literature [6]. 

*Friedelin* (**6**): White needles, m.p. 245–246 °C (lit. [7] 260–263 °C). Spectral data agreed with the literature [7]. 

*Betulinic acid* (**7**): White solid, m.p. 290–291 °C (lit. [8] 291–292 °C). Spectral data were consistent with published data [9,10].

## 4. Conclusions

Two new pyranoxanthones—mesuaferrin A (**1**) and mesuaferrin B (**2**)—were isolated from the root bark of *Mesua ferrea*. along with one other xanthone, one anthraquinone, and three triterpenes. This is the first report on the phytochemistry of the root bark of *Mesua ferrea* and the data reported here should contribute to the database of the chemistry of *Mesua* species.

## References

[1] Verotta L., Lovaglio E., Vidari G., Finzi P.V., Neri M.G., Raimondi A., Parapini S., Taramelli D., Riva A., Bombardelli E. (2004). 4-Alkyl- and 4-phenylcoumarins from *Mesua ferrea* as promising multidrug resistant antibacterials. Pytochemistry.

[2] Ee G.C.L., Lim C.K., Ong G.P., Sukari M.A., Lee H.L. (2006). Daphnifolin, a new xanthone from *Mesua daphnifolia*. J. Asian Nat. Prod. Res..

[3] Teh S.S., Ee G.C.L., Rahmani M., Sim W.C., Mah S.H., Teo S.H. (2010). Two New Pyranoxanthones from *Mesua beccariana* (Guttiferae). Molecules.

[4] Iinuma M., Tosa H., Tanaka T., Yonemori S. (1994). Two new xanthones in the underground part of *Calophyllum inophyllum*. Heterocycles.

[5] Ee G.C.L., Kua A.S.M., Rahmani M. (2007). Anthraquinones and xanthones from *Cratoxylum glaucum* (Guttiferae). Pertanika J. Sci. Technol..

[6] Jamaluddin F., Mohamed S., Lajis M.N. (1994). Hypoglycaemic effect of *Parkia speciosa* seeds due to the synergistic action of beta-sitosterol and stigmasterol. Food Chem..

[7] Ee G.C.L., Ng K.N., Yap Y.H.T., Rahmani M., Ali A.M., Muse R. (2004). Mucigerin, A new coumarin from *Calophyllum mucigerum* (Guttiferae). Nat. Prod. Res..

[8] Kim D.S.H.L., Chen Z., Nguyen V.T., Pezzuto J.M., Qiu S., Lu Z. (1997). A concise semi-synthetic approach to betulinic acid from betulin. Synth. Commun..

[9] Galgon T., Hoke D., Drager B. (1999). Identification and Quantification of Betulinic Acid. Phytochem. Anal..

[10] Tapondjou A.L., Miyamoto T., Dubois M.A.L. (2006). Glucuronide Triterpene Saponins from *Bersama engleriana*. Phytochemistry.

